# Epigenetic Regulation of Ferroptosis in Chronic Kidney Disease: Mechanisms and Implications

**DOI:** 10.34133/research.0934

**Published:** 2025-10-09

**Authors:** Zi-Hui Mao, Yong Liu, Qing Zhang, Shaokang Pan, Duo Chen, Yingjin Qiao, Hui Wang, Dongwei Liu, Zhangsuo Liu, Qi Feng

**Affiliations:** ^1^ Department of Nephrology, the First Affiliated Hospital of Zhengzhou University, Zhengzhou University, Zhengzhou 450052, P. R. China.; ^2^ Research Institute of Nephrology, Zhengzhou University, the First Affiliated Hospital of Zhengzhou University, Zhengzhou 450052, P. R. China.; ^3^ Traditional Chinese Medicine Integrated Department of Nephrology, the First Affiliated Hospital of Zhengzhou University, Zhengzhou 450052, P. R. China.; ^4^ Henan Province Research Center for Kidney Disease, the First Affiliated Hospital of Zhengzhou University, Zhengzhou 450052, P. R. China.; ^5^ Key Laboratory of Precision Diagnosis and Treatment for Chronic Kidney Disease in Henan Province, the First Affiliated Hospital of Zhengzhou University, Zhengzhou 450052, P. R. China.; ^6^ Tianjian Laboratory of Advanced Biomedical Sciences, Academy of Medical Sciences, Zhengzhou University, Zhengzhou, Henan 450001, P. R. China.; ^7^ Innovation Center of Basic Research for Metabolic-Associated Fatty Liver Disease, Ministry of Education of China, Zhengzhou, P. R. China.; ^8^ Department of Endocrinology and Metabolism, the First Affiliated Hospital of Zhengzhou University, Zhengzhou 450052, P. R. China.; ^9^ Blood Purification Center, the First Affiliated Hospital of Zhengzhou University, Zhengzhou 450052, P. R. China.

## Abstract

Chronic kidney disease (CKD) is a prevalent and progressive condition that leads to renal structural abnormalities and a gradual decline in kidney function. CKD has various etiologies, including diabetes, hypertension, and glomerulonephritis, and is associated with significant morbidity, mortality, and economic burden. Current treatments focus on slowing disease progression and managing complications; however, CKD often progresses to end-stage renal disease, necessitating renal replacement therapy. Therefore, innovative therapeutic approaches are urgently required. Recent studies have highlighted the role of ferroptosis, an iron-dependent form of cell death characterized by lipid peroxidation and oxidative stress, in CKD pathogenesis. Ferroptosis contributes to structural damage and functional impairment in renal cells. Furthermore, epigenetic modifications, including DNA methylation and histone changes, regulate gene expression without altering the DNA sequence and have been implicated in CKD progression. These epigenetic alterations may influence inflammation, fibrosis, and ferroptosis, thereby exacerbating renal dysfunction. This review explores the intersection of ferroptosis and epigenetic regulation in CKD, offering novel insights into the mechanisms driving disease progression and potential therapeutic targets. Through a comprehensive bibliometric analysis, this study provides a deeper understanding of CKD pathogenesis and proposes potential future treatment strategies.

## Introduction

Chronic kidney disease (CKD) is a prevalent, complex, and progressive condition characterized by renal structural abnormalities and gradual loss of kidney function over time [1]. Clinical studies have confirmed that CKD predominantly arises from diverse pathogenic factors, including diabetes, hypertension, glomerulonephritis, polycystic kidney disease, and exposure to certain toxins or drugs. Current treatment strategies for CKD are aimed primarily at slowing disease progression and preventing complications, such as cardiovascular disease, anemia, and mineral–bone disorders, while emphasizing a healthy lifestyle, optimal control of blood glucose and blood pressure, and effective management of associated complications [2]. Without timely treatment or proper management, CKD may evolve into end-stage renal disease (ESRD). The morbidity and fatality rates associated with CKD are on a consistent rise annually, making it a prominent concern in global public health [2]. Therefore, novel therapeutic approaches to slow disease progression and improve patient prognosis are urgently needed.

Ferroptosis is an iron-dependent, reactive oxygen species (ROS)-accumulating, lipid peroxidation-driven form of programmed cell death [3]. Unlike apoptosis, necrosis, and other common types of cell death, ferroptosis primarily involves the dysregulation of iron metabolism and lipid peroxidation, and the disruption of antioxidant systems [4]. Ferroptosis serves as a significant contributor to the pathogenesis and progression of various diseases. In the context of CKD, the mechanisms of ferroptosis and its impact on disease progression have emerged as central focuses of recent studies [5]. Investigating ferroptosis is crucial for understanding CKD pathogenesis. Specifically, this research aimed to determine whether ferroptosis occurs in both CKD animal models and human patients. Furthermore, studies have sought to establish the contribution of ferroptosis to disease initiation and progression and clarify its detrimental effects on renal cells, such as structural damage and functional impairment [6]. Moreover, studies have focused on identifying specific factors that influence the occurrence and progression of CKD through ferroptosis modulation.

**Fig. 1.**
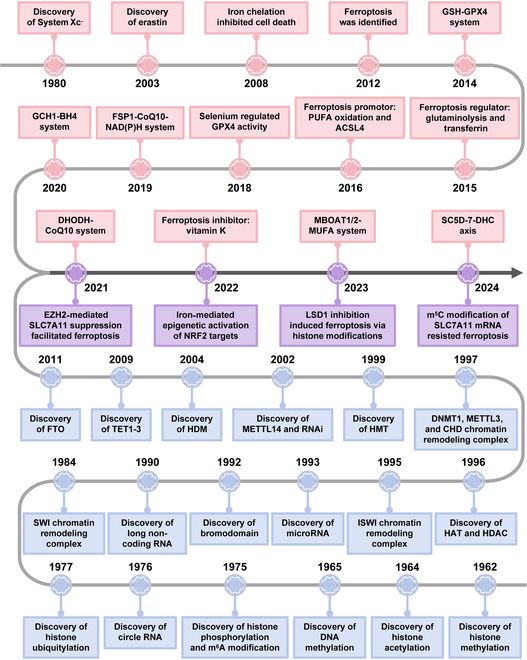
Historical timeline of key discoveries related to ferroptosis and epigenetic regulation. This figure chronologically maps pivotal milestones in ferroptosis research (pink) and epigenetic modification studies (blue), highlighting their parallel evolution and interdisciplinary intersections. The upper half highlights discoveries related to ferroptosis mechanisms, including iron metabolism, lipid peroxidation, and antioxidant systems. The lower half maps key developments in epigenetics, such as DNA methylation, histone modifications, and noncoding RNA discoveries. The interconnected nature of these fields underscores their combined importance in understanding cellular processes and disease mechanisms. System Xc^−^, cystine–glutamate antiporter system; GSH, glutathione; GPX4, glutathione peroxidase 4; GCH1, guanosine triphosphate cyclohydrolase-1; BH4, tetrahydrobiopterin; FSP1, ferroptosis suppressor protein-1; CoQ10, coenzyme Q10; NAD(P)H, nicotinamide adenine dinucleotide phosphate; PUFA, peroxidation of polyunsaturated fatty acid; ACSL4, acyl-coenzyme A synthetase long-chain family member 4; DHODH, dihydroorotate dehydrogenase; MBOAT1/2, membrane bound O-acyltransferase domain containing 1/2; MUFA, monounsaturated fatty acid; SC5D, sterol-C5-desaturase; 7-DHC, 7-dehydrocholesterol; EZH2, enhancer of zeste homolog 2; SLC7A11, solute carrier family 7 member 11; Nrf2, nuclear factor erythroid 2-related factor 2; LSD1, lysine specific demethylase 1; m^5^C, 5-methylcytosine; FTO, fat mass and obesity-associated protein; TET1-3, ten-eleven translocation 1-3; HDM, histone demethylase; METTL14, methyltransferase like 14; RNAi, RNA interference; HMT, histone-by-histone methyltransferase; DNMT1, DNA methyltransferase 1; METTL3, methyltransferase like 3; CHD, chromodomain helicase DNA-binding; HAT, histone acetyltransferase; HDAC, histone deacetylase; m^6^A, N^6^-methyladenosine.

Epigenetic modifications refer to the regulation and modification of genes that influence their expression and function without altering their DNA sequence. They encompass DNA methylation, histone modifications, and noncoding RNA-mediated epigenetic regulation [7]. Epigenetic modifications play critical roles in life processes by determining which genes are activated or silenced at specific times and in specific tissues, thereby affecting processes such as cell differentiation, development, and metabolism [8]. The importance of epigenetic modifications lies in their ability to finely regulate gene expression patterns without modifying the genetic code, thereby influencing cellular functions and fates [9]. In CKD, aberrant epigenetic modifications may affect the expression of genes involved in processes such as inflammation, extracellular matrix (ECM) deposition, tubular epithelial cell transdifferentiation, and ferroptosis, thereby contributing to disease progression [10]. This review article systemically discusses the roles of ferroptosis and epigenetic modifications in CKD and explores how epigenetically regulated ferroptosis affects disease occurrence and progression. Figure 1 illustrates key milestones in the research on ferroptosis and epigenetic modification. Additionally, a bibliometric analysis of academic research on epigenetic modifications, ferroptosis, and CKD was conducted. This review aims to offer novel perspectives on etiopathogenesis and the advancement of therapeutic strategies for CKD.

## Bibliometric Analysis of Epigenetic Modifications and Ferroptosis in CKD

An extensive literature search was conducted via the Web of Science Core Collection from 2012 January 1 (the year the concept of ferroptosis was first introduced), to 2024 November 1. To ensure the inclusion of all relevant studies, a detailed search strategy incorporating a range of synonymous terms was employed, as outlined in Table S1. The search was restricted to publications classified as “Articles” or “Review Articles” and written in English, yielding 810 articles on ferroptosis, epigenetic modifications, and CKD. The data were then analyzed and visualized via Microsoft Office Excel (version 2021), SCImago Graphica (version 1.0.46), VOSviewer (version 1.6.20), CiteSpace (version 6.3.1), and the R package “bibliometrix”.

**Fig. 2.**
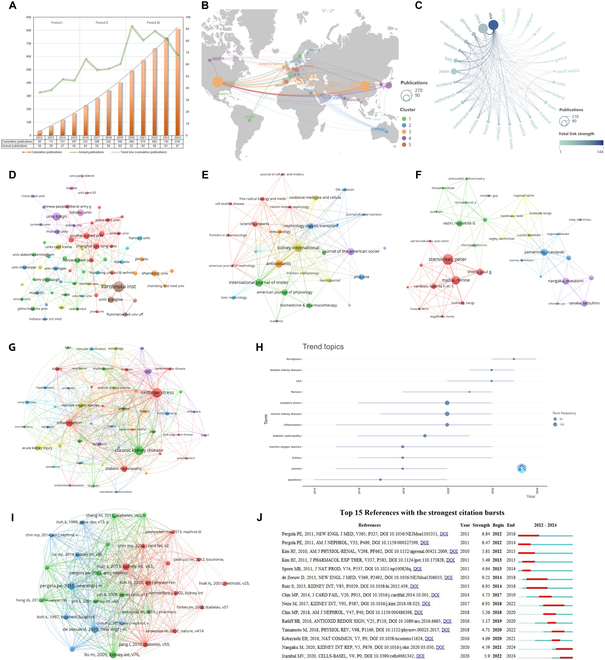
Bibliometric analysis of epigenetic modifications and ferroptosis in CKD. (A) Temporal trends in annual and cumulative publications (2012–2024), demonstrating a marked acceleration in research output over the past 5 years. (B) Global distribution of contributions, with circle size reflecting publication volume and color intensity denoting collaboration density. (C) Country collaboration network dominated by the United States and China. (D) Institutional collaboration network, revealing regionally clustered partnerships despite international engagement. (E) Citation relationships among journal sources. (F) Author collaboration network highlighting active cooperation among researchers. (G) Keyword co-occurrence map identifying core themes. (H) Keyword evolution timeline underscoring the increasing importance of ferroptosis in CKD research. (I and J) Co-citation network and citation burst analysis of influential references, offering insights into key literature and their interconnections and emphasizing the role of ferroptosis-related transcription factors (e.g., Nrf2) in CKD pathogenesis.

Figure 2A presents a line graph depicting the annual number of publications, along with histograms representing the cumulative publication counts. The data demonstrate a steady increase in publications over the past decade, marked by a significant acceleration in the most recent 5 years. Figure 2B illustrates the global distribution and interconnections among contributing countries, where the size of each circle corresponds to the publication volume, and the color indicates the intensity of cooperative activity. Furthermore, visualization networks were constructed (Fig. 2C) to represent publication counts and collaborative relationships among countries. The analysis highlights that international collaboration is predominantly centered around the United States and China, with link strengths ranked first (144) and second (61), respectively. The top 10 most productive countries are presented in Table S2.

Institutional collaborations were visualized in a network (Fig. 2D), with the top 10 most productive organizations listed in Table S3. The findings revealed that although international partnerships exist, institutional collaborations are predominantly domestic and regionally concentrated. Figure 2E displays active citation relationships among journal sources, with the top 10 most cited journals summarized in Table S4. Moreover, the visualization of author collaborations (Fig. 2F) indicates relatively active cooperation between researchers. Among the top 10 most productive authors (Table S5), Peter Stenvinkel and Denise Mafra were identified as the leading contributors.

Co-occurrence analysis of authors’ keywords revealed that “oxidative stress”, “inflammation”, and “nuclear factor erythroid 2-related factor 2 (Nrf2)” constitute the primary research focuses in the domain of ferroptosis and epigenetic modifications in CKD (Fig. 2G and Table S6). Keyword trend analysis recently highlighted the increasing prominence of ferroptosis as a central theme in CKD research (Fig. 2H). Additionally, the cocited reference network and citation burst analyses provided valuable insights into the most frequently cited literature in this area and the interconnections among these references (Fig. 2I and J). Notably, the majority of the 15 most-cited references emphasized the role of ferroptosis-related transcription factors in CKD pathogenesis (Table S7). Among these factors, the transcription factor Nrf2 effectively mitigates oxidative stress and inflammation, thereby offering protection against kidney damage [11].

## Ferroptosis and CKD

**Fig. 3.**
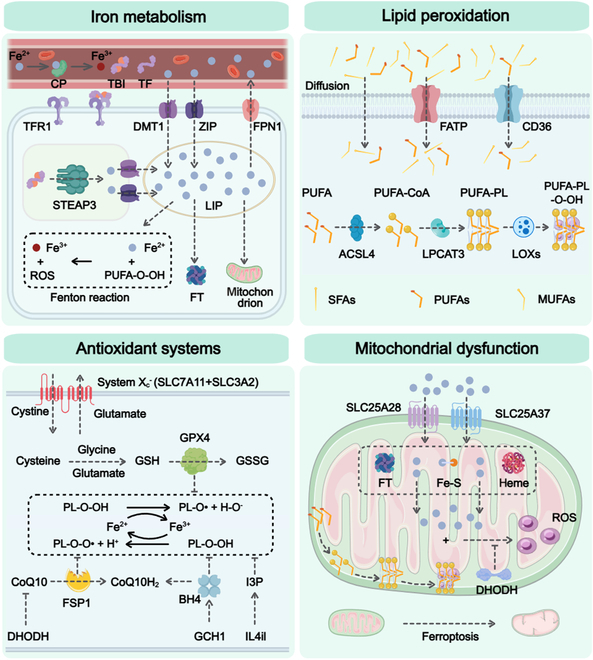
Molecular mechanisms underlying ferroptosis. This schematic delineates the core pathways driving ferroptosis, which are organized into 4 interconnected modules. Iron homeostasis is regulated through iron uptake, reduction, and storage. LIP dynamics involve Fe^2+^/Fe^3+^ cycling via DMT1 and FPN1. Excess Fe^2+^ catalyzes Fenton reactions, generating ROS that initiate lipid peroxidation. PUFAs are esterified by ACSL4 and incorporated into membrane phospholipids via LPCAT3. LOXs oxidize PUFA-PLs to peroxidized derivatives (PUFA-PL-O-OH), promoting membrane destabilization. Saturated fatty acids and monounsaturated fatty acids antagonize peroxidation. The GPX4–GSH axis neutralizes lipid peroxides (PL–O–OH), whereas the FSP1–CoQ10H2 and DHODH–CoQ10H2 systems provide parallel redox protection. System X_c_^−^ (SLC7A11/SLC3A2) maintains cysteine availability for GSH synthesis. IL4i1 modulates ferroptosis via I3P signaling. Mitochondrial iron influx (via SLC25A37/28) exacerbates ROS production. Disrupted electron transport chains and cardiolipin oxidation amplify oxidative stress, whereas DHODH supports CoQ10_H2_-mediated antioxidant defense. The figure was created with MedPeer (medpeer.cn). CP, ceruloplasmin; TBI, transferrin-bound iron; TF, transferrin; TFR1, transferrin receptor-1; DMT1, divalent metal transporter-1; ZIP, ZRT/IRT-like protein; FPN-1, ferroportin-1; STEAP3, six-transmembrane epithelial antigen of prostate 3; LIP, labile iron pool; ROS, reactive oxygen species; FT, ferritin; FATP, fatty acid transport protein; PL, phospholipids; LPCAT3, lysophosphatidylcholine acyltransferase 3; LOXs, lipoxygenases; SFAs, saturated fatty acids; MUFAs, monounsaturated fatty acids; System Xc^−^, cystine–glutamate antiporter system; SLC3A2, solute carrier family 3 member 2; GSH, glutathione; GSSG, oxidized glutathione; IL4i1, interleukin-4-induced-1; I3P, indole-3-pyruvate; SLC25A37, solute carrier family 25 member 37; SLC25A28, solute carrier family 25 member 28.

**Fig. 4.**
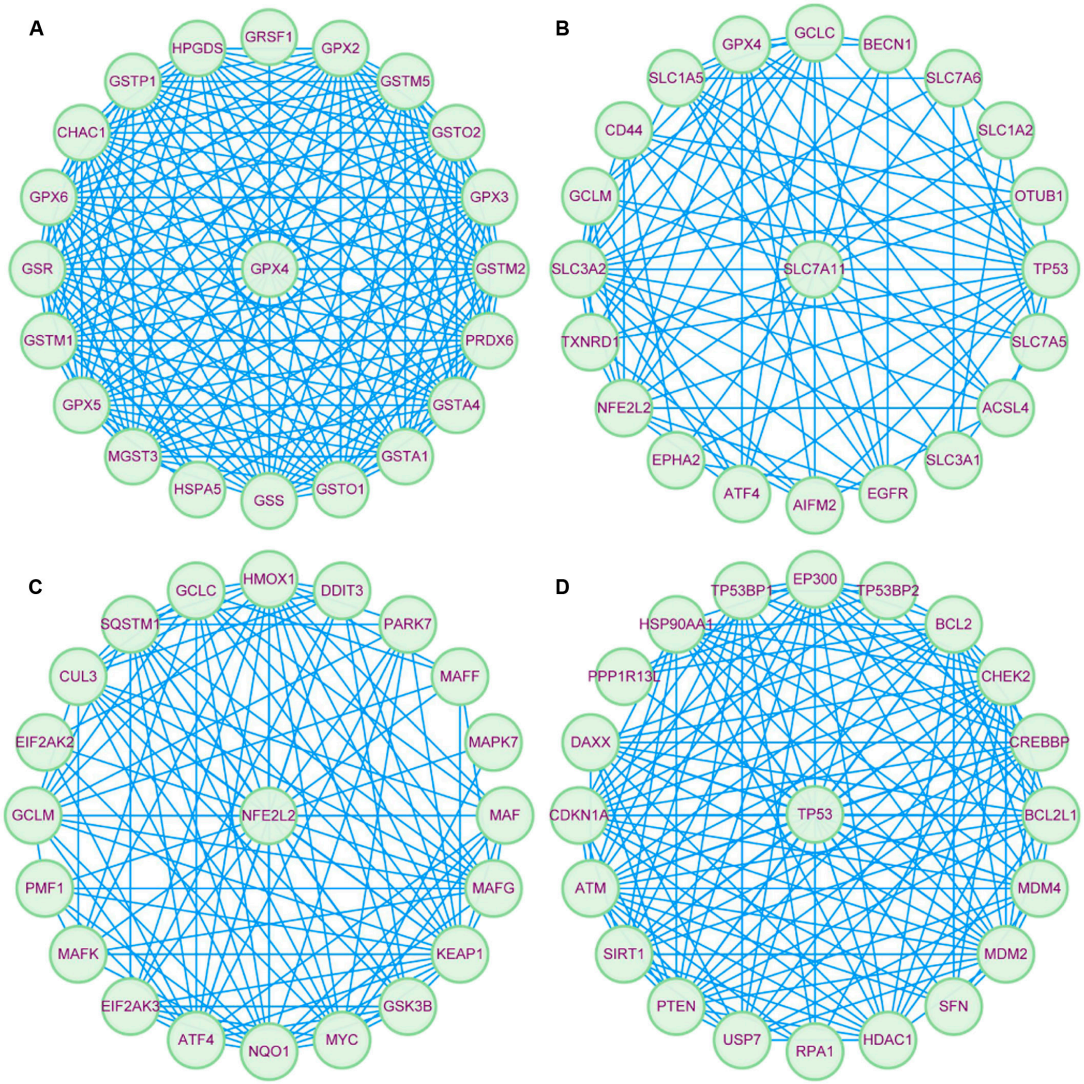
Protein–protein interaction network of ferroptosis-related regulators and target genes. The interaction networks for GPX4 (A), SLC7A11 (B), NFE2L2 (C) and TP53 (D) with their target genes were generated by using the STRING database and visualized in Cytoscape. This network map integrates multilayered regulation of ferroptosis, spanning redox balance, iron–lipid dynamics, and epigenetic–metabolic crosstalk, providing a systems-level perspective for therapeutic discovery. GRSF1, G-rich sequence factor 1; GPX2, glutathione peroxidase 2; GSTM5, glutathione S-transferase Mu 5; GSTO2, glutathione S-transferase omega-2; GPX3, glutathione peroxidase 3; GSTM2, glutathione S-transferase Mu 2; PRDX6, peroxiredoxin-6; GSTA4, glutathione S-transferase A4; GSTA1, glutathione S-transferase A1; GSTO1, glutathione S-transferase omega-1; GSS, glutathione synthetase; HSPA5, heat shock protein family A (hsp70) member 5; MGST3, microsomal glutathione S-transferase 3; GPX5, glutathione peroxidase 5; GSTM1, glutathione S-transferase Mu 1; GSR, glutathione reductase; GPX6, glutathione peroxidase 6; CHAC1, ChaC glutathione specific gamma-glutamylcyclotransferase 1; GSTP1, glutathione S-transferase Pi 1; HPGDS, hematopoietic prostaglandin D synthase; GCLC, glutamate-cysteine ligase catalytic subunit; BECN1, beclin-1; SLC7A6, solute carrier family 7 member 6; SLC1A2, solute carrier family 1 member 2; OTUB1, ubiquitin thioesterase OTUB1; TP53, tumor protein 53; SLC7A5, solute carrier family 7 member 5; SLC3A1, solute carrier family 3 member 1; EGFR, epidermal growth factor receptor; AIFM2, flavoprotein apoptosis-inducing factor mitochondrial 2; ATF4, activating transcription factor 4; EPHA2, ephrin type-A receptor 2; NFE2L2, nuclear factor erythroid 2-related factor 2; TXNRD1, thioredoxin reductase 1; GCLM, glutamate-cysteine ligase modifier subunit; SLC1A5, solute carrier family 1 member 5; HMOX1, heme oxygenase 1; DDIT3, DNA damage-inducible transcript 3 protein; PARK7, parkinson disease protein 7; MAFF, MAF basic leucine zipper transcription factor F; MAPK7, mitogen-activated protein kinase 7; MAF, MAF BZIP transcription factor; MAFG, MAF BZIP transcription factor G; KEAP1, kelch-like ECH-associated protein 1; GSK3B, glycogen synthase kinase-3 beta; MYC, MYC proto-oncogene; NQO1, NAD(P)H quinone dehydrogenase 1; EIF2AK3, eukaryotic translation initiation factor 2 α kinase 3; MAFK, MAF BZIP transcription factor K; PMF1, polyamine modulated factor 1; EIF2AK2, eukaryotic translation initiation factor 2 α kinase 3; CUL3, cullin 3; SQSTM1, sequestosome 1; EP300, EP300 lysine acetyltransferase; TP53BP2, tumor protein 53 binding protein 2; BCL2, BCL2 apoptosis regulator; CHEK2, checkpoint kinase 2; CREBBP, CREB binding lysine acetyltransferase; BCL2L1, BCL2 like 1; MDM4, mouse double minute 4; MDM2, murine double minute 2; SFN, Stratifin; HDAC1, histone deacetylase 1; RPA1, replication protein A1; USP7, ubiquitin specific peptidase 7; PTEN, phosphatase and tensin homolog; SIRT1, sirtuin 1; ATM, ATM serine/threonine kinase; CDKN1A, cyclin-dependent kinase inhibitor 1A; DAXX, death domain associated protein; PPP1R13L, protein phosphatase 1 regulatory subunit 13 like; HSP90AA1, heat shock protein 90 α family class A member 1; TP53BP1, tumor protein 53 binding protein 1.

Ferroptosis, which is characterized by iron-dependent lipid peroxidation-driven membrane damage, may be hindered by glutathione peroxidase 4 (GPX4). This mechanism was first proposed by Dixon et al. [3] in 2012 and was confirmed in erastin-induced cancer cells. Ferroptosis tends to be initiated in cells exhibiting disrupted iron and thiol redox metabolism, and the kidneys are particularly susceptible to redox imbalances. Growing evidence suggests that ferroptosis is linked to CKD caused by various factors, such as diabetic nephropathy (DN), hypertensive nephropathy, and glomerulonephritis [4]. Oxidative stress and mitochondrial dysfunction, which are hallmarks of ferroptosis, can also trigger apoptotic signaling pathways, indicating potential crosstalk. Moreover, the release of damage-associated molecular patterns during ferroptosis may promote necroinflammatory responses, thereby exacerbating necrosis [12]. These interactions suggest that ferroptosis not only acts independently but also may amplify or modulate other cell death pathways, contributing to the progressive kidney injury observed in CKD. In the subsequent sections, we highlight the molecular mechanisms of ferroptosis (Fig. 3) and ferroptosis-related regulators and target genes (Fig. 4) involved in CKD.

## Key Mechanisms of Ferroptosis

Ferroptosis, a regulated form of cell death driven by iron-dependent lipid peroxidation, is a pivotal mechanism underlying cellular dysfunction and disease progression. The molecular underpinnings of this condition involve 4 interconnected axes: dysregulation of iron metabolism, lipid peroxidation cascades, antioxidant system failure, and mitochondrial damage. These pathways collectively orchestrate oxidative membrane disruption and organelle collapse, with implications for conditions such as CKD.

### Iron metabolism

Iron, a vital trace element in the human body, fulfills a diverse range of important biological roles. Homeostasis of intracellular iron metabolism involves iron uptake, storage, release, and recycling processes [3]. Under physiological conditions, cells take up iron via transferrin (TF)- and transferrin receptor (TFR)-mediated endocytosis. In the bloodstream, Fe^2+^ is oxidized to Fe^3+^ by ceruloplasmin (CP), which binds to TF to form transferrin-bound iron (TBI) [13]. TBI enters cells through the membrane protein TFR-1. Fe^3+^ is transported into the labile iron pool (LIP) through divalent metal transporter-1 (DMT1) or ZRT/IRT-like protein (ZIP), and is then reduced to Fe^2+^ by the six-transmembrane epithelial antigen of prostate 3 (STEAP3) [13]. In the cytoplasm, iron from the LIP can be utilized by the mitochondria, whereas excess Fe^2+^ is stored in ferritin (FT) in a protein-bound form. FT converts Fe^2+^ to Fe^3+^ and exports it out of the cell through ferroportin-1 (FPN-1), maintaining cytoplasmic iron homeostasis [13]. The release of iron from the FT can be mediated by factors such as Nrf2, whose activation induces the expression of the Nrf2-associated antioxidant proteins heme oxygenase-1 (HO-1) and FT. These proteins help protect human podocytes from oxidative damage induced by hemoglobin endocytosis [14].

Excess free Fe^2+^ reacts with hydrogen peroxide (H_2_O_2_) in the Fenton reaction to produce highly reactive hydroxyl radicals (·OH). Fe^2+^ can also activate ferroptosis-related enzymes, such as lipoxygenases (LOXs), further promoting the peroxidation of polyunsaturated fatty acids (PUFAs) in cell membrane [15]. Consequently, increased iron uptake and decreased iron efflux increase cellular sensitivity to oxidative damage and ferroptosis. Knocking down the key regulatory factor of ferritinophagy, nuclear receptor coactivator 4 (NCOA4), mitigates autophagy-induced ferroptosis [16]. Owing to the limited affinity of TF-binding sites for iron ions, elevated tissue iron concentrations may exceed the TF-binding capacity. Unbound iron ions are bound primarily to serum albumin and citrate, forming nontransferrin-bound iron (NTBI) [17]. During iron recycling, NTBI readily participates in redox reactions, such as the Fenton and Haber–Weiss reactions, generating ·OH. The kidney is highly sensitive to iron, and its absorption and clearance mechanisms are distinct from those of the reticuloendothelial system, predisposing it to iron deposition and overload in tissues [18]. In patients with CKD, iron deposition has been detected in the proximal and distal tubules of the kidneys, in contrast to healthy controls [19]. While the specific role of iron in ferroptosis is still under investigation, it is critical for lipid peroxidation and ferroptosis.

### Lipid peroxidation

Lipid peroxidation is an oxidative reaction involving lipids in biological systems and is initiated by free radicals. Compared with monounsaturated and unsaturated fatty acids, PUFAs are more susceptible to lipid peroxidation and are essential for the execution of ferroptosis [3]. Lipid peroxidation disrupts cell membrane structure, affects normal cellular metabolism and physiological functions, and is strongly associated with the progression of CKD [6]. Lipid peroxidation involves multiple steps, including free radical generation, lipid molecule oxidation, and the accumulation of oxidative products. The peroxidation process is a self-propagating chain reaction. When a free radical oxidizes a PUFA molecule, a lipid radical forms [6]. This lipid radical reacts with oxygen to form a lipid peroxyl radical. This radical can extract a hydrogen atom from another PUFA molecule, continuing the chain reaction and leading to lipid peroxide accumulation [20]. The products of lipid peroxidation chain reactions are highly bioactive, are capable of damaging DNA and proteins, exhibit high enzymatic bioactivity, and serve as molecular signals that activate cell death [21]. Among PUFA-associated phospholipids, acyl-arachidonoyl (AA) and adrenoyl (AdA) are the primary substrates in the lipid peroxidation process. The severity of ferroptosis is determined by the localization and abundance of PUFAs.

Free PUFAs, as substrates for synthesizing lipid signaling mediators, must be esterified by activated acyl-coenzyme A synthetase long-chain family member 4 (ACSL4) and incorporated into membrane phospholipids via lysophosphatidylcholine acyltransferase 3 (LPCAT3). These proteins are subsequently oxidized by LOXs to generate ferroptosis signals [12]. The regulation of enzymes associated with the biosynthesis of PUFAs within membrane phospholipids can either initiate or inhibit ferroptosis. Conversely, the lack of ACSL4 and LPCAT3 can hinder ferroptosis induced by GPX4 inhibitors such as RSL3 and ML162 [22]. LOXs catalyze the addition of molecular oxygen to PUFAs, producing lipid peroxides. LOX inhibitors, such as liproxstatin-1 and vitamin E, effectively suppress ferroptosis [23]. In summary, a deeper understanding of the mechanisms by which lipid peroxidation induces ferroptosis is critical for developing novel therapeutic strategies and preventive measures.

### Antioxidant systems

The antioxidant system involves a series of mechanisms by which organisms combat oxidative stress, including various antioxidant enzymes and nonenzymatic antioxidants. The GPX4–glutathione (GSH) antioxidant system serves as a key protective mechanism against ferroptosis [4]. GPX4, a selenoprotein, uses GSH as a cofactor to reduce lipid peroxides to their corresponding alcohols. By reducing lipid peroxides to their corresponding alcohols, GPX4 halts lipid peroxidation propagation and protects cells from ferroptosis, placing GPX4 at the core of ferroptosis regulation [24]. GSH is an intracellular antioxidant that inhibits oxidative stress caused by oxidants such as H₂O₂ and suppresses the accumulation of lipid ROS [25]. GSH depletion increases cellular susceptibility to ferroptosis, whereas GSH synthesis endows cells with resistance. GSH synthesis depends on the availability of cysteine, which is obtained via the cystine–glutamate antiporter system (system Xc^−^) [4]. System Xc^−^, a heterodimer, consists of solute carrier family 7 member 11 (SLC7A11) and solute carrier family 3 member 2 (SLC3A2), which are widely distributed in cell membranes [3]. System Xc^−^ transports cystine into cells, where it is reduced to cysteine for GSH synthesis. Therefore, various factors that cause intracellular GSH depletion and GPX4 degradation regulate sensitivity to ferroptosis. For example, ferroptosis-inducing factor-56 and chaperone-mediated autophagy promote ferroptosis by facilitating GPX4 degradation [26].

In addition to the GPX4–GSH antioxidant system, there are other GPX4-independent systems that modulate ferroptosis. These include ferroptosis suppressor protein-1 (FSP1)/coenzyme Q10 (CoQ10) [27], guanosine triphosphate cyclohydrolase-1 (GCH1)/tetrahydrobiopterin (BH4) [28], dihydroorotate dehydrogenase (DHODH) [29], and amino acid oxidase interleukin-4-induced-1 (IL4i1)/indole-3-pyruvate (I3P) [30]. CoQ10 is a lipophilic antioxidant that scavenges free radicals. FSP1, a flavoprotein oxidoreductase, uses nicotinamide adenine dinucleotide phosphate [NAD(P)H] to catalyze the reduction of CoQ10 to CoQ10H_2_. This enzymatic reaction efficiently mitigates the propagation of lipid peroxides, thus suppressing ferroptosis. The GCH1-derived metabolite BH4, a lipophilic antioxidant, prevents ferroptosis by specifically blocking the consumption of phospholipids with 2 polyunsaturated acyl chains, thereby inducing lipid remodeling [28]. Increased levels of BH4 also reduce oxidative damage by depleting CoQ10 and PUFAs. DHODH, located in the inner mitochondrial membrane, plays a key role in catalyzing the synthesis of pyrimidine nucleotides. DHODH is a flavoprotein that reduces CoQ10. In the process of oxidizing dihydroorotate to orotate, DHODH transfers electrons to CoQ10, reducing it to CoQ10H_2_ [29]. IL4i1 suppresses ferroptosis by producing I3P from tryptophan, triggering free radical scavenging, and activating antioxidant gene expression programs for cellular protection [30]. These antioxidant systems work in conjunction with the GPX4–GSH system to maintain redox balance and prevent excessive accumulation of lipid peroxides.

### Mitochondrial dysfunction

Mitochondria, which are enclosed by a double membrane, play a central role in energy generation, metabolic regulation, cell survival, and tissue renewal. Ferroptosis is characterized by prominent changes in mitochondrial morphology, including shrinkage, increased membrane density, and the loss or reduction of mitochondrial cristae [3]. The mitochondrial membrane, abundant in PUFAs, is highly susceptible to lipid peroxidation. Lipid peroxidation in mitochondria disrupts the potential of the mitochondrial membrane and electron transport chain (ETC) functions. ETC disruption results in increased ROS production, creating a vicious cycle of oxidative damage and mitochondrial dysfunction [31].

One of the important contributors to ferroptosis is the accumulation of iron within the mitochondria. The mitochondrial import of extracellular iron occurs via transporters, including solute carrier family 25 member 37 (SLC25A37) and solute carrier family 25 member 28 (SLC25A28), which are similar to plasma membrane transporters [32]. In mitochondria, Fe^2+^ is typically used for heme and Fe–S cluster synthesis or is stored in the mitochondrial FT. Excessive Fe^2+^ accumulation in the mitochondria can generate ROS through Fenton reactions [33]. ROS damage the mitochondrial membrane and increase enzyme activity, further exacerbating mitochondrial dysfunction. The mitochondrial iron balance is closely linked to the transport of key redox metabolites, such as GSH, within the mitochondria [32]. Additionally, some mitochondria-associated proteins are implicated in ferroptosis. For example, cardiolipin, a mitochondria-specific phospholipid, is oxidized during ferroptosis. Cardiolipin oxidation affects the function of mitochondrial proteins, such as cytochrome c oxidase and adenosine triphosphate (ATP) synthase, leading to energy metabolism disorders and cell death [34]. Interest in the relationship between organelles and ferroptosis is increasing; nonetheless, the exact molecular pathways through which mitochondria contribute to ferroptosis remain unclear.

### Ferroptosis-related genes involved in CKD

In CKD, specific genes and transcription factors closely associated with ferroptosis are likely to modulate ferroptosis through their effects on iron metabolism, lipid peroxidation, or antioxidant systems [6]. Aberrant expression or alterations in the functionality of these genes potentially increase the susceptibility of renal cells to ferroptosis, thus facilitating CKD progression. GPX4 serves as the core modulator of ferroptosis. Under normal physiological circumstances, GPX4 is essential for sustaining cell membrane stability by inhibiting lipid peroxide accumulation [35]. In CKD, a reduction in GPX4 expression and activity results in increased lipid peroxidation, thereby increasing the susceptibility of renal cells to ferroptosis. SLC7A11, a subunit of system Xc^−^, is involved in the intracellular transport of cystine. Given that SLC7A11 supplies cystine for GSH synthesis and that intracellular GSH levels are essential for maintaining GPX4 activity, any perturbation in SLC7A11 function directly affects GPX4 activity, and the converse is also true [36].

Nrf2 is an important transcription factor involved in modulating the expression of a diverse array of antioxidant- and detoxification-related genes, including FT, system Xc^−^, and GPX4. By orchestrating these gene networks, Nrf2 is essential for maintaining cellular metabolism and redox equilibrium [37]. Under typical physiological conditions, Nrf2 is confined to the cytoplasm and is expressed at low levels. Nevertheless, upon exposure to oxidative stress or other ferroptosis-inducing stimuli, Nrf2 is liberated and translocated into the nucleus. Moreover, it interacts with antioxidant response elements (AREs) to initiate the transcription of genes implicated in antioxidant defense, such as GPX4 and SLC7A11 [38]. In CKD, Nrf2 may influence ferroptosis through multiple signaling pathways. Dysfunctional Nrf2 activation may lead to inadequate up-regulation of genes associated with protection against ferroptosis [18]. Conversely, in certain circumstances, overactivation of Nrf2 may have adverse consequences. For example, in advanced CKD with sustained oxidative stress, prolonged Nrf2 activation may lead to a compensatory but ultimately ineffective response, allowing ferroptosis [39].

TP53, a tumor suppressor gene, is strongly linked to ferroptosis. It transcriptionally regulates the expression of genes that play a role in cystine uptake and oxidative stress. Consequently, maintaining TP53 at a stable basal level can prevent ferroptosis [40]. In addition to the genes described above, other genes involved in regulating ferroptosis, such as ACSL4, NCOA4, and HO-1 [41], contribute to CKD pathogenesis. Therefore, understanding the roles of these genes in CKD is critical for identifying potential therapeutic strategies.

## Epigenetic Modifications Involved in CKD

**Fig. 5.**
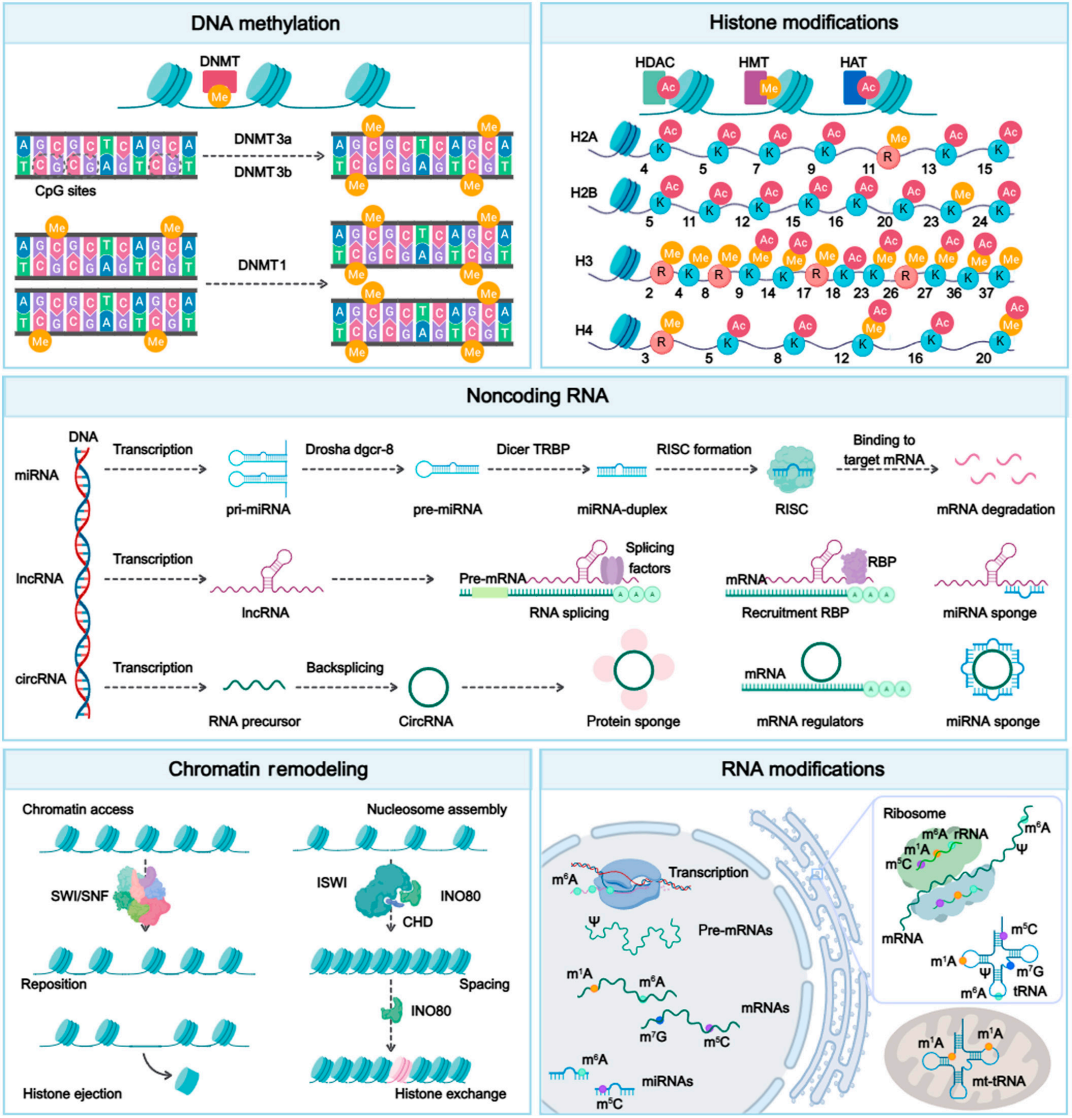
Potential epigenetic mechanisms contributing to CKD. This figure provides a comprehensive overview of the diverse epigenetic mechanisms involved in CKD. The process of DNA methylation at CpG sites, which is mediated by DNMTs (including DNMT1, DNMT3a, and DNMT3b), can lead to gene silencing or activation, influencing CKD progression. Various histone modifications, including acetylation, methylation, and phosphorylation, are catalyzed by enzymes such as HDACs, HMTs, and HATs. These modifications influence chromatin structure and gene transcription, thereby contributing to the pathogenesis of CKD. The roles of miRNAs, lncRNAs, and circRNAs are critical in regulating gene expression through mechanisms such as mRNA degradation, RNA splicing, and acting as miRNA sponges. These regulatory processes markedly influence the development of CKD. The action of chromatin remodeling complexes, such as SWI/SNF, ISWI, CHD, and INO80, involves the repositioning, ejection, or restructuring of nucleosomes, thereby modulating gene accessibility and expression in the context of CKD. Various RNA modifications, including m^6^A, m^5^C, and Ψ, influence RNA stability, splicing, translation, and other facets of gene expression, thereby contributing to the pathophysiology of CKD. The figure was created with MedPeer (medpeer.cn). DNMT, DNA methyltransferase; miRNAs, microRNAs; lncRNAs, long noncoding RNAs; circRNAs, circular RNAs; RISC, RNA-induced silencing complex; mRNA, messenger RNA; RBP, RNA binding protein; SWI/SNF, switch/sucrose nonfermentable; ISWI, imitation switch; m^1^A, N^1^-methyladenosine; Ψ, pseudouridine; m^7^G, N^7^-methylguanosine; tRNA, transfer RNA; mt-tRNA, mitochondrial-transfer RNA.

Recently, researchers have discovered that epigenetic modifications are becoming increasingly important in CKD progression. These modifications can modulate gene expression without changing the DNA sequence. DNA methylation can silence or activate genes, whereas histone modifications can change chromatin structure, thus influencing gene transcriptional activity. Noncoding RNAs, including microRNAs (miRNAs), long noncoding RNAs (lncRNAs), and circular RNAs (circRNAs), are capable of regulating gene expression at the epigenetic level through interactions with target genes [8]. Accordingly, understanding the underlying epigenetic mechanisms of CKD is essential for demonstrating the intricate pathophysiology of the disease and identifying possible therapeutic targets (Fig. 5).

### DNA methylation in CKD

DNA methylation refers to a covalent modification where a methyl group is added to the fifth carbon of cytosine residues within CpG islands, which are DNA regions rich in CpG dinucleotides. DNA methylation is usually linked to the repression of genes, which inhibits gene transcription and thereby affects gene expression levels [7]. This process is catalyzed by DNA methyltransferases (DNMTs), such as DNMT1, DNMT3A, and DNMT3B. DNMT1 plays a key role in preserving methylation patterns during DNA replication, whereas DNMT3A and DNMT3B are primarily responsible for de novo methylation [42]. Normal kidneys exhibit specific DNA methylation patterns and generally low methylation levels, whereas abnormal methylation, particularly hypermethylation, has been observed in various kidney diseases [43]. A study of the DNA methylation profiles of 443 kidneys revealed approximately 140,000 methylated CpG sites, and the heritability mediated by DNA methylation was found to be highly tissue-specific [44]. A genome-wide epigenomic association study from a Hong Kong prospective cohort revealed 48 CpG sites near genes with important functional roles in kidney diseases, highlighting the potential of methylation markers in risk stratification in type 2 diabetes patients with kidney conditions [45]. In addition, the methylation of several genes is strongly associated with the progression of CKD. For example, hypermethylation in the promoter region of the nephrin gene, which is involved in kidney function and metabolism, can reduce the expression of nephrin and podocyte Kruppel-like factor 4 (KLF4), thereby affecting the integrity of the glomerular filtration barrier and leading to proteinuria [46]. Genes involved in ECM production and cell cycle regulation may exhibit different methylation patterns. Hypomethylation of genes encoding collagen and fibronectin can result in their overexpression, promoting excessive ECM accumulation and the development of fibrosis [47]. Overall, focusing on DNA methylation offers a potential therapeutic strategy for CKD.

### Histone modifications in CKD

Histones are fundamental structural proteins that constitute chromosomes, and various modifications can occur, including methylation, acetylation, phosphorylation, crotonylation, and ubiquitination [9]. These modifications take place on the amino-terminal tails of histones (H2A, H2B, H3, and H4), which make up the nucleosome. Histone acetylation is usually related to gene activation, as it neutralizes the positive charge of histones, resulting in a more accessible chromatin structure that facilitates the binding of transcription factors to DNA and enhances gene transcription [48]. Conversely, histone deacetylation is typically associated with gene repression. Histone acetylation and deacetylation are catalyzed by histone acetyltransferases (HATs) and histone deacetylases (HDACs), respectively [49]. Histone methylation involves the transfer of a methyl group to a lysine or arginine residue on histone-by-histone methyltransferases (HMTs), and its effects vary depending on the specific residue and methylation level. For example, H3K4 methylation is generally associated with gene activation [50], whereas H3K9 methylation may cause chromatin condensation and inhibit gene transcription [51]. Thus, histone modifications can regulate the expression of genes involved in CKD pathophysiology, thereby playing a critical role in controlling gene transcription. Experiments have demonstrated that progressive glomerulosclerosis related to DN is associated with H3K9 acetylation and H3K4 methylation in the kidney [52]. H3K27 is highly methylated in renal fibroblasts cultured from unilateral ureteral obstruction (UUO) mice and CKD patients, as well as in fibrotic kidneys. This modification activates profibrotic signaling pathways by down-regulating the expression of Smad7 and tensin homolog (PTEN), thereby mediating renal fibrosis in CKD [53]. After renal ischemia–reperfusion injury, a reduction in HAT activity leads to decreased histone acetylation and lower expression of bone morphogenetic protein-7 in proximal tubule cells, increasing the susceptibility of renal cells to damage [54]. Elevated histone acetylation of proinflammatory and profibrotic genes following acute kidney injury (AKI) facilitates the progression from AKI to CKD [55]. These results emphasize the heterogeneity of histone acetylation in AKI. Furthermore, a notable reduction in H3K27 methylation in the podocytes of *db/db* mice leads to the up-regulation of pathological factors associated with podocyte damage, thereby disrupting podocyte homeostasis and promoting glomerular damage [56].

### Noncoding RNA-mediated epigenetic regulation in CKD

Noncoding RNAs include miRNAs, lncRNAs, and circRNAs. miRNAs are small noncoding RNAs approximately 20 to 25 nucleotides in length. They regulate gene expression by binding to the 3′ untranslated region (3′-UTR) of target messenger RNAs (mRNAs), leading to mRNA degradation or inhibition of translation [57]. A study revealed that miRNAs in circulating small extracellular vesicles play a critical role in CKD-driven vascular calcification [58]. Elevated miR-34a expression in renal tubular epithelial cells substantially down-regulates the expression of Klotho, an endogenous renal fibrosis inhibitor, thereby promoting fibrotic processes [59]. Additionally, another miRNA, miR-192, is specifically expressed in the kidneys and is involved in the regulation of renal fibrosis and glomerular function. Dysregulation of miR-192 alters the expression of genes related to glomerular basement membrane components, ECM deposition, and cell differentiation [60].

LncRNAs are noncoding RNAs exceeding 200 nucleotides in length that interact with DNA, RNA, or proteins to regulate gene expression. They can regulate the expression of nearby genes in cis by interacting with the chromatin of adjacent genes and modulating the chromatin state. LncRNAs can also function in trans, regulating genes located on different chromosomes [61]. Moreover, lncRNAs can interact with the spliceosome complex, affecting the splicing patterns of precursor mRNAs and producing distinct mRNA isoforms with different functions. Similar to miRNAs, lncRNA-mediated posttranscriptional regulation has been shown to restore Klotho expression, inhibit cellular senescence, and suppress renal fibrosis [62]. LncRNAs also contribute in various ways to the progression from AKI to CKD at the transcriptional and translational levels. Research has suggested that the down-regulation of lncRNA-PVT1 may be linked to CKD progression in patients with congestive heart failure [63]. Furthermore, lncRNA H19 contributes to CKD-related glomerulosclerosis and tubulointerstitial fibrosis through diverse mechanisms, including epigenetic, posttranscriptional, and posttranslational regulation, as well as the induction of inflammatory responses, apoptosis, ferroptosis, pyroptosis, autophagy, and oxidative damage [64].

CircRNAs are a class of noncoding RNAs characterized by covalently closed-loop structures. They contain multiple binding sites for miRNAs, and by sequestering these miRNAs, circRNAs can prevent them from interacting with their target mRNAs, thereby providing a posttranscriptional regulatory mechanism that finely tunes gene expression [65]. Growing evidence suggests that circRNAs are crucial in the regeneration and repair of renal tubular cells following kidney injury and in the progression from AKI to CKD. Genome-wide circRNA analyses in rats with hypertension revealed that numerous circRNAs are differentially expressed in the rat kidneys of hypertensive nephropathy model, indicating their potential involvement in the pathogenesis of kidney injury [66]. One study reported that the overexpression of the circRNA itchy E3 ubiquitin protein ligase alleviates oxidative stress and mitochondrial dysfunction in septic AKI by targeting miR-214-3p [67]. In addition, expression profiling of circRNAs in DN patients revealed 40 up-regulated and 23 down-regulated circRNAs in the glomeruli and renal tubules. The expression levels of the hub genes targeted by these circRNAs are correlated with renal function, highlighting the crucial regulatory roles of circRNAs in glomerular and tubular functions in DN [68].

### Chromatin remodeling in CKD

Chromatin remodeling is a fundamental mechanism that regulates gene expression by dynamically altering chromatin structure. Nucleosomes, the basic units of chromatin, can be repositioned, ejected, or restructured by chromatin remodeling complexes that utilize ATP hydrolysis to modify the chromatin structure [69]. Chromatin remodeling complexes can be classified into 4 main families: switch/sucrose nonfermentable (SWI/SNF), imitation switch (ISWI), chromodomain helicase DNA-binding (CHD), and INO80, each with distinct enzymatic functions and regulatory roles in chromatin remodeling [70]. Chromatin remodeling plays a critical role in kidney development and function. During nephrogenesis, remodeling complexes regulate genes critical for nephron formation and cellular differentiation, including *Pax2* and *WT1*, ensuring their expression at appropriate developmental stages and locations [71]. In mature kidneys, chromatin remodeling continues to regulate genes involved in glomerular filtration, tubular reabsorption, and secretion, enabling the kidney to respond to physiological fluctuations in blood pressure, fluid and electrolyte homeostasis, and metabolic changes [72].

In CKD, chromatin remodeling is crucial in driving fibrosis, inflammation, and cellular senescence. Chromatin remodeling complexes can interact with transcription factors and histone-modifying enzymes, thereby influencing the transcription of genes related to ECM production [73]. Dysregulation of the SWI/SNF complex contributes to the up-regulation of genes linked to ECM accumulation and epithelial–mesenchymal transition (EMT) in renal tubular cells [74]. In response to kidney injury, the chromatin remodeling protein Brahma-related gene 1 (BRG1) modifies the chromatin landscape surrounding cytokine gene loci, thereby increasing their expression and amplifying the inflammatory response [75]. This sustained inflammatory signaling further exacerbates renal damage. Additionally, chromatin remodeling contributes to the up-regulation of genes by altering chromatin accessibility at gene loci. This promotes the secretion of fibrosis- and inflammation-related factors, ultimately driving CKD progression.

Chromatin remodeling also intricately interacts with other epigenetic mechanisms in CKD, including DNA methylation and histone modification. DNA methylation affects the recruitment of chromatin-remodeling complexes, as methylated CpG islands serve as binding sites for chromatin-remodeling proteins, thereby modulating their interactions with DNA [76]. Conversely, chromatin remodeling shapes DNA methylation patterns by altering chromatin accessibility. These complexes can expose or occlude CpG islands by repositioning nucleosomes, thereby affecting their methylation status [77]. Histone acetylation enhances chromatin accessibility and facilitates the recruitment of chromatin remodeling complexes to specific genomic regions, promoting transcriptionally permissive chromatin states. Chromatin remodeling can influence histone modification patterns by modifying nucleosome positioning, which affects the accessibility of histone-modifying enzymes to their target sites [78]. Taken together, these dynamic interactions underscore the complexity of epigenetic regulation in patients with CKD. A deeper understanding of chromatin remodeling and its crosstalk with other epigenetic mechanisms may reveal novel therapeutic targets for mitigating CKD progression.

### RNA modifications in CKD

RNA modifications refer to the chemical alterations in RNA molecules that serve as crucial regulatory layers in gene expression and various biological processes. These modifications can occur through 2 primary mechanisms: RNA-independent and RNA-guided pathways. In RNA-independent mechanisms, modifications are catalyzed directly by specific enzymes, whereas RNA-guided mechanisms involve the cooperative action of guide RNAs and protein complexes, including modifying enzymes [79]. Several types of RNA modifications have been identified, the most common of which include N^6^-methyladenosine (m^6^A), 5-methylcytosine (m^5^C), pseudouridine (Ψ), and N4-acetylcytidine (ac4C) [80].

m^6^A is the most common internal modification of mRNAs, is installed by the m^6^A methyltransferase complex, and is removed by specific demethylases. This modification influences several aspects of mRNA metabolism, such as splicing, export, stability, and translation. Under certain conditions, m^6^A can recruit specific RNA binding proteins (RBPs), thereby enhancing or inhibiting mRNA translation [81]. Dysregulation of the expression of m^6^A methyltransferases or demethylases results in aberrant m^6^A levels in mRNAs associated with kidney function and disease progression. For example, inhibiting the m^6^A-associated methyltransferase METTL3 has been shown to alleviate renal fibrosis [82]. METTL3 enhances the stability of TAB3 through m^6^A modification, and its suppression mitigates kidney injury and inflammation via an IGF2BP2-dependent pathway [83]. Knockdown of METTL3 also decreases m^6^A methylation of MDM2, thereby preventing mitochondrial damage and ferroptosis in renal tubular epithelial cells following AKI [84]. Furthermore, m^6^A modification is involved in the regulation of genes associated with CKD inflammation. The m^6^A methylation of nucleotide-binding oligomerization domain, leucine-rich repeat, and pyrin domain-containing protein 3 (NLRP3) promotes the activation of the NLRP3 inflammasome, thereby inducing pyroptosis and inflammation [85].

m^5^C modification primarily occurs in transfer RNA (tRNA) and ribosomal RNA (rRNA), although it is also present in mRNAs. In tRNA, m^5^C is vital for preserving the correct tertiary structure and stability, which are essential for accurate protein translation [80]. Loss of tRNA m^5^C modification can lead to translation errors in transport proteins involved in electrolyte balance or enzymes involved in metabolic pathways. m^5^C modification influences mRNA export from the nucleus and its stability, thereby regulating gene expression at the posttranscriptional level [86]. For example, m^5^C modification induced by TRDMT-1 in B cells can regulate immunoglobulin A (IgA) class switching, and inhibition of m^5^C modification has been shown to improve the progression of IgA nephropathy in mouse models [87].

Ψ modification involves rotation and isomerization of the N–C glycosidic bond of uridine, forming Ψ. This modification occurs in various RNAs. It alters the spatial structure of RNA, affecting the interactions between RNA molecules and between RNA and proteins. Ψ modification by PUS7 impacts translation initiation complex-mediated translation regulation through tRNA-derived small fragments, guiding translation control in stem cells and protein biosynthesis [88]. In rRNA, Ψ modification is essential for ribosome assembly and prioritizes the translation of beneficial proteins. The optimization of the ribosomal structure is critical for efficient protein synthesis. Ψ modification in RNA is catalyzed by the H/ACA small nucleolar ribonucleoprotein (snoRNP) complex, and mutations in its core proteins DKC1 and NOP10 lead to reduced Ψ modification in rRNA, causing ribosomal dysfunction and a genetic disease characterized by telomere attrition, which clinically manifests as nephrotic syndrome [89]. In CKD, RNA modifications interact with other epigenetic processes, including histone modifications and noncoding RNAs. The m^6^A-related methyltransferase METTL14 alleviates renal tubular EMT by increasing PTEN expression and down-regulating HDAC5, thereby reducing ECM accumulation in kidneys of diabetic mouse [90]. By regulating the expression of tRNA aspartate methyltransferase 1, miR-23b influences TRDMT-1-driven m^5^C RNA modification and serum IgA regulation [87]. Accordingly, understanding RNA modifications in CKD can provide new insights into disease mechanisms and potential therapeutic targets.

## Epigenetic Modification-Regulated Ferroptosis in CKD

**Fig. 6.**
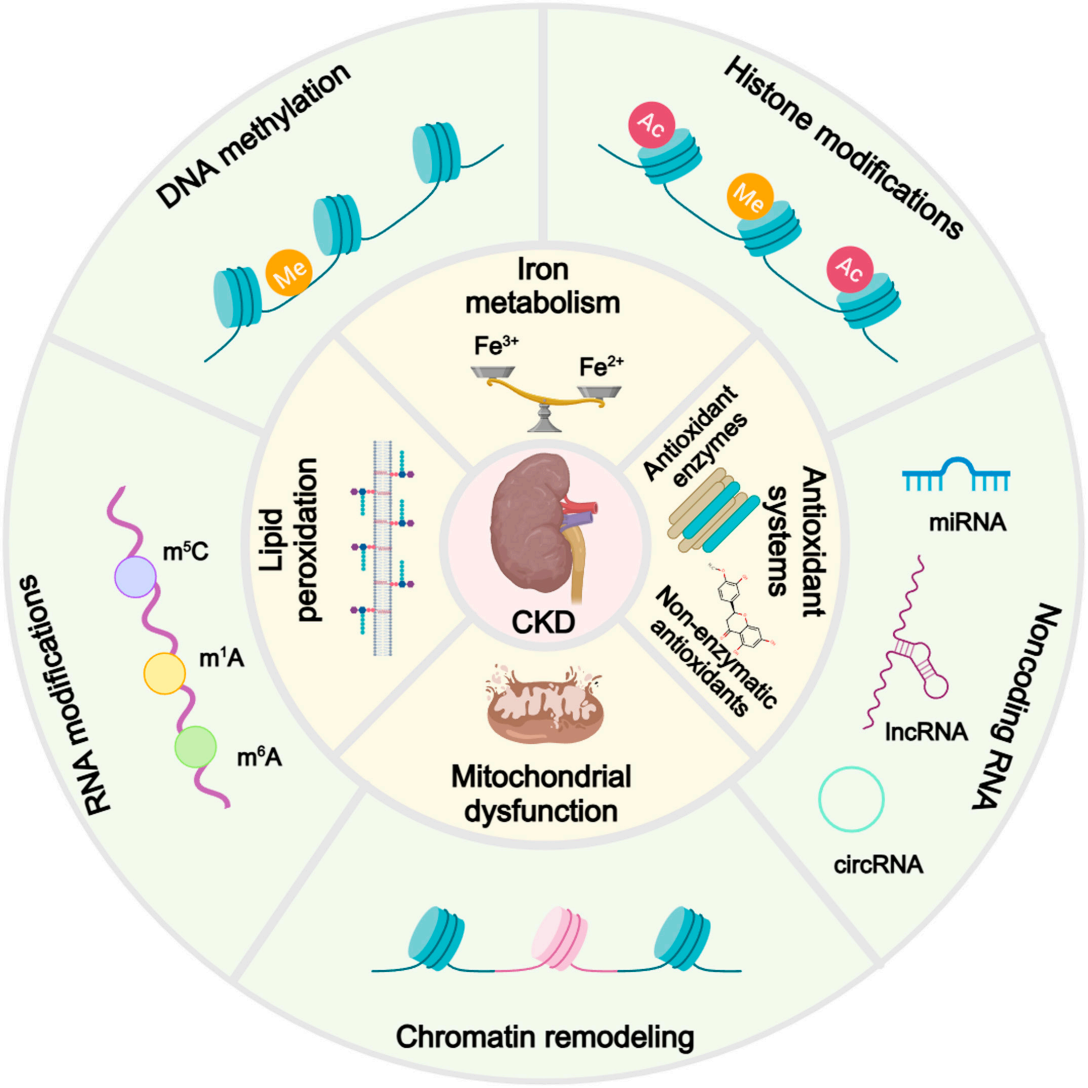
Epigenetic regulation of ferroptosis in CKD. Persistent epigenetic–ferroptosis interplay exacerbates tubular injury, inflammation, and fibrosis, culminating in CKD progression. The figure was created with MedPeer.

Epigenetic modifications control gene expression without changing the DNA sequence and are crucial in the regulation of ferroptosis [91] (Fig. 6). DNA methylation influences the expression of genes involved in ferroptosis, and abnormal DNA methylation patterns linked to ferroptosis have been detected in the kidneys [92]. Histone modifications affect the expression of genes related to iron metabolism, lipid peroxidation, and antioxidant defense pathways during ferroptosis [93]. For example, HDAC3 interacts with the GPX4 promoter, causing local histone hypoacetylation, which suppresses GPX4 transcription, induces renal ferroptosis, and accelerates the progression of AKI to CKD [94]. HMT G9a interacts with the transcription factor Bach1 and inhibits the transcription of SLC7A11 through H3K9 dimethylation, thereby activating ferroptosis. Therefore, inhibiting G9a reduces inflammatory cytokine production and collagen deposition in UUO mice, making it an attractive target for treating renal fibrosis [95]. Similarly, EZH2 facilitates ferroptosis in calcium oxalate-induced kidney injury by down-regulating SLC7A11 expression via H3K27 trimethylation [96]. Furthermore, knockdown of the RNA modification-associated methyltransferase METTL3 reduces MDM2 m^6^A methylation, decreases intracellular iron ion and ROS levels, alleviates mitochondrial damage, and increases the levels of SOD, GSH, GPX4, FPN-1, and TFR1, thus shielding renal tubular epithelial cells from mitochondrial injury and ferroptosis after AKI [84].

Additionally, miRNAs and lncRNAs can target ferroptosis-related genes and regulate their expression, thus playing a role in the epigenetic regulation of ferroptosis in CKD. In cisplatin-induced AKI, miR-214-3p targets GPX4 and exacerbates ferroptosis, whereas inhibition of miR-214-3p enhances the expression of GPX4 and SLC7A11 and reduces ACSL4 expression, providing protective effects against ferroptosis and renal tubular damage [97]. Specific lncRNAs can act as miRNA “sponges”, targeting ferroptosis-related genes to modulate their expression. For example, lncRNA H19 acts as a “sponge” for miR-129, regulating oxidative stress and ferroptosis triggered by hyperglycemia in mesangial cells via the Nrf2 pathway and contributing to glomerulosclerosis in DN [98]. Herbal treatments, such as *Astragalus mongholicus* Bunge and Panax notoginseng, can alleviate renal fibrosis in UUO mice by down-regulating lncRNA A33 expression and inhibiting ferroptosis [99]. Notably, different epigenetic mechanisms are not independent but exhibit complex crosstalk in ferroptosis regulation. Epigenetic modifications and posttranslational modifications, as well as their mutual regulation, can affect specific genomic loci, such as the promoters of GPX4 [100] or SLC7A11 [101], thereby fine-tuning the regulation of ferroptosis [10]. Although studies on the epigenetic regulation of ferroptosis in CKD are still limited, understanding the complex pathophysiological mechanisms of CKD and developing new therapeutic methods are crucial. In the future, increasing attention is expected to be given to this area.

## Therapeutic Implications

An increasing number of studies are examining the impact of ferroptosis and epigenetic modifications on CKD and have explored whether epigenetic regulation of ferroptosis could be a potential therapeutic target for CKD. Strategies to prevent ferroptosis-mediated kidney injury include blocking lipid peroxidation and ROS accumulation [41,102] by using iron chelators [103,104], enhancing GPX4 activity or SLC7A11 expression [105], and modulating Nrf2-activating compounds [106]. For example, ferrostatin-1 and 16-86 (a novel third-generation ferrostatin) inhibit ferroptosis in the kidneys by preventing lipid peroxidation and ROS accumulation [41,102]. Liproxstatin-1 not only inhibits lipid peroxidation but also reduces iron deposition [107]. Necrostatin-1 (Nec-1) and Nec-1f, small-molecule inhibitors of receptor-interacting protein kinase 1 (RIPK1), suppress ROS accumulation and exert a mild inhibitory effect on ferroptosis [102,108]. Peroxisome proliferator-activated receptor γ (PPAR-γ) agonists, including rosiglitazone, pioglitazone, and troglitazone, inhibit ACSL4 expression and down-regulate ferroptosis-induced renal inflammatory responses and fibrosis [109,110]. Ginkgolide B [111] and astragaloside-IV [112] up-regulate GPX4 expression by inhibiting GPX4 ubiquitination and suppressing the hypoxia-inducible factor-1α (HIF-1α)/HO-1 pathway, respectively, exerting effects similar to those of platycodin D [113], which also inhibits ferroptosis. Notably, discrepancies in drug efficacy across different CKD models may be attributed to several factors, including differences in disease stage (e.g., acute versus chronic models), underlying etiology (e.g., DN versus ischemia–reperfusion injury), and species or strain variation (e.g., mouse versus rat models). These factors can prominently influence the expression of ferroptosis-related genes, cellular oxidative status, and therapeutic responses. Table 1 summarizes the ferroptosis-related strategies for CKD treatment.

**Table 1.** Strategies for CKD treatment related to ferroptosis

Reagents	Models	Mechanisms	References
Ferrostain-1	*Pkd1* mutant mouse models	Inhibition of lipid peroxidation and ROS accumulation	[41]
Liproxstatin-1	UUO mouse model	Inhibition of lipid peroxidation and iron deposition	[107]
Forsythoside A	DN-related databases	Inhibition of ROS accumulation	[152]
Nec-1	RIPK3-deficient mice	RIPK1 inhibitor (inhibits ROS accumulation)	[102]
Nec-1f	*Gpx4^cys/−^* mice, *Fsp1*-deficient mice	A solid inhibitor of RIPK1 and a weak inhibitor of ferroptosis (unknown target)	[108]
Deferoxamine mesylate	5/6 nephrectomy-induced CKD rat model	Iron chelators	[103]
Deferasirox	5/6 nephrectomy-induced CKD rat model	Iron chelators	[103]
Rosiglitazone	AngII-induced hypertension mouse model	ACSL4 inhibitor	[110]
Pioglitazone	*Gpx4^−/−^* mice	ACSL4 inhibitor	[109]
Troglitazone	*Gpx4^−/−^* mice	ACSL4 inhibitor	[109]
Quercetin	DN mouse model	Up-regulated the GPX4 and SLC7A11 expression, activated Nrf2	[105]
Platycodin D	High glucose-induced HK-2 cells	Up-regulated GPX4 expression	[113]
Ginkgolide B	DN mouse model	Up-regulated GPX4 expression (inhibited GPX4 ubiquitination)	[111]
Astragaloside-IV	DN mouse model and high glucose-induced HK-2 cells	Up-regulated GPX4 expression (inhibited HIF-1α/HO-1 pathway)	[112]
Tectorigenin	UUO mouse model	Restored GPX4 expression	[153]
Bardoxolone methyl	Human CKD	Activated Nrf2	[106]
Curcumin	Human CKD	Activated Nrf2	[106]
Resveratrol	Human CKD	Activated Nrf2	[106]
Canagliflozin	DN mouse model	Improved fatty acid oxidation	[154]

ROS, reactive oxygen species; Nec-1, necrostatin-1; RIPK1, receptor-interacting protein kinase 1; ACSL4, acyl-coenzyme A synthetase long-chain family member 4; GPX4, glutathione peroxidase 4; SLC7A11, the cystine/glutamate reverse antiporter solute carrier family 7 member; Nrf2, nuclear factor erythroid 2-related factor 2; HIF-1α, hypoxia-inducible factor-1α; HO-1, heme oxygenase 1; MCTR1, maresin conjugates in tissue regeneration 1; FSP1, ferroptosis suppressor protein-1; *Gpx4^cys/−^*, *Gpx4* targeted *cys* mutation; *Gpx4*^−/−^, *Gpx4* knockout

Epigenetic drugs, including DNMT inhibitors, HMT inhibitors, and HDAC inhibitors, can reverse abnormal epigenetic patterns in CKD. 5-Azacytidine, a DNMT1 inhibitor, reduces renal inflammation and proteinuria via the phosphatase and PTEN and VDR signaling axes by inhibiting hypermethylation of the PTEN promoter and VDR [114]. Two other DNMT inhibitors, decitabine and dihydroartemisinin, inhibit renal fibrosis in mice by reducing the methylation levels of NDRG2 and Klotho promoters, respectively [115,116]. Similarly, blocking HMTs has an antifibrotic effect. For example, EPZ5676, AZ505, sinefungin, and BIX01294 inhibit the lysine methyltransferases DOT1L, SMYD2, SET7/9, and G9a, respectively, reversing the abnormal methylation patterns of H3K79, H3K36, H3K4, and H3K9, and alleviating renal fibrosis [117–120]. The selective PRMT1 inhibitor AMI reduces the asymmetric dimethylation of histone H4 arginine 3 (H4R3Me2a), inhibiting renal fibroblast activation and fibrosis [121].

Additionally, class I and II HDAC inhibitors, including vorinostat, trichostatin A, and FR276457, have been studied for their potential to improve renal function by regulating histone acetylation and gene expression [122–124]. Tubastatin A, a highly selective HDAC6 inhibitor, alleviates cisplatin-induced renal dysfunction [125]. Valproic acid and 4-methyl-thiobutanate, as class I HDAC inhibitors, promote the repair of doxorubicin- and aristolochic acid-induced kidney injury and reduce postinjury fibrosis [126,127]. In addition to histone methylation and acetylation, histone crotonylation plays a role in mitigating DN-related inflammation and fibrotic damage and restoring renal function [128]. Moreover, inhibitors of bromodomain and extraterminal motif (BET) proteins, such as MS417, have been found to alleviate kidney injury [129]. Nevertheless, additional studies are needed to refine these drugs, including determining appropriate dosages, treatment durations, and patient selection criteria, to ensure the safety and efficacy of epigenetics-based therapies. Table 2 summarizes the strategies related to DNA methylation and histone modifications for CKD.

**Table 2.** Strategies for CKD treatment related to DNA methylation and histone modifications

Reagents	Models	Mechanisms	References
5-Azacytidine	HBV-GN mouse model	DNMT inhibitor (inhibited the DNMT1 expression, regulated PTEN and VDR signaling axis)	[114]
Decitabine	UUO mouse model	DNMT inhibitor (corrected the abnormal DNMT1/3A/3B expression, reduced the methylation level of NDRG2 promoter)	[115]
Dihydroartemisinin	UUO and adenine-induced mouse models	DNMT inhibitor (reduced the DNMT1 expression and reversed Klotho promoter hypermethylation)	[116]
AZ505	UUO mouse model	HMT inhibitor (inhibited SMYD2 and H3K36me3)	[118]
Sinefungin	UUO mouse model	HMT inhibitor (inhibited SET7/9 and H3K4me1)	[119]
BIX01294	UUO mouse model	HMT inhibitor (inhibited G9a and H3K9me1)	[120]
EPZ5676	UUO mouse model	HMT inhibitor (inhibited DOT1L and H3K79 methylation)	[118]
AMI-1	UUO mouse model	HMT inhibitor (inhibited PRMT1 and H4R3Me2a)	[121]
OICR-9429	I/R injury mouse model	HMT inhibitor (inhibited MLL1/WDR5 and H3K4me3)	[155]
Vorinostat	*Col4a3*^−*/*−^ mice	HDAC inhibitor (attenuated JNK phosphorylation and reduced MAPK activation)	[122]
Trichostatin A	Cisplatin-induced kidney injury	HDAC inhibitor (up-regulated AMWAP expression)	[123]
Valproic acid	Adriamycin-induced kidney injury	HDAC inhibitor (inhibited HDAC1)	[126]
M4PTB	Aristolochic acid-induced kidney injury	HDAC inhibitor (inhibited HDAC1)	[127]
Tubastatin A	Cisplatin-induced kidney injury	HDAC inhibitor (inhibited HDAC6)	[125]
FR276457	UUO rat model	HDAC inhibitor (inhibited MCP-1 production)	[124]
Crotonate	DN mouse model and high glucose-induced HK-2 cells	Induced histone Kcr and H3K18cr	[128]
MS417	DN mouse model	BET inhibitor (blocked acetylation-mediated association of p65 and STAT3 with BET proteins)	[129]

DNMT, DNA methyltransferase; HDAC, histone deacetylase; NDRG2, N-myc downstream-regulated gene 2; AMWAP, activated microglia/macrophage WAP domain protein; PTEN, phosphatase and tensin homolog; VDR, vitamin D receptor; HMT, histone methyltransferase; SMYD2, SET and MYND domain protein 2; H3K36me3, histone H3 lysine 36 trimethylation; SET7/9, SET domain-containing lysine methyltransferase; H3K4me1, histone H3 lysine 4 monomethylation; H3K9me1, histone H3 lysine 9 monomethylation; MAPK, mitogen-activated protein kinases; M4PTB, 4-methyl-thiobutanate; MCP-1, monocyte chemotactic protein 1; H3K18cr, histone H3 lysine 18 crotonylation; Kcr, histone lysine crotonylation; BET, bromodomain and extraterminal; STAT3, signal transducer and activator of transcription 3; DOT1L, disruptor of telomeric silencing-1 like; H3K79, histone H3 lysine 79; PRMT1, protein arginine methyltransferase 1; H4R3Me2a, histone 4 arginine 3 asymmetric di-methylated; MLL1, mixed-lineage leukemia 1; WDR5, WD-40 repeat protein 5; H3K4me3, histone 3 lysine 4 trimethylation; HBV-GN, hepatitis B virus-associated glomerulonephritis; *Col4a3*^−*/*−^, *Col4a3* knockout

The application of noncoding RNAs, such as miRNA antagonists, mimics, and anti-miRNA oligonucleotides, to block the function of miRNAs that promote ferroptosis could lead to promising therapeutic strategies. For instance, antagonists of miR-214 have been found to exert protective effects on kidneys after injury [130]. The silencing of miR-132 has also demonstrated potential for treating renal fibrosis [131]. Anti-miR-21 oligonucleotides improve mitochondrial function and protect against fibrosis and inflammation in glomerular and interstitial cells by inhibiting miR-21 [132]. When overexpressed, miR-192 mimics enhance collagen matrix expression through transforming growth factor-β (TGF-β)/Smad3 signaling, whereas the addition of miR-192 inhibitors can prevent TGF-β/Smad3-induced fibrosis [60]. In contrast, miR-181a mimics alleviate kidney injury, whereas miR-181a inhibitors worsen kidney damage [133]. Moreover, the miR-200b precursor and exosome-encapsulated miR-26a are potential novel therapeutic targets for improving tubulointerstitial fibrosis and treating kidney disease [134,135]. However, as miRNAs are regulated in a manner that is not specific to cell types or organs, targeting them may lead to unintended effects in unrelated organs, presenting challenges for the clinical application of miRNA-based therapies. Table 3 summarizes the miRNA-based strategies for treating CKD.

**Table 3.** Strategies for CKD treatment related to miRNAs

Reagents	Models	Mechanisms	References
Anti-miR-214	UUO mouse model	miR-214 (independent of TGF-β pathway)	[130]
Antagomir-132	UUO FoxD1-GC; Z/Red-mice model	Silenced miR-132 (decreased myofibroblast proliferation)	[131]
Anti-miR-21 oligonucleotides	Alport nephropathy mouse model	Silenced miR-21 (enhanced PPARα/RXR activity and improved mitochondrial function)	[132]
miR-181a mimic/inhibitor	Rats with CKD	miR-181a (down-regulation of the CRY1 gene and the TLR/NF-κB pathway)	[133]
miR-200b precursor	UUO mouse model	Produced miR-200b (ameliorated tubulointerstitial fibrosis in obstructed kidneys)	[134]
Exo-miR-26a	Aldosterone-induced kidney injury	Overexpressed miR-26a (decreased expression of connective tissue growth factor and SMAD3 inhibition)	[135]

miRNA, microRNA; UUO, unilateral ureteral obstruction; TGF-β, transforming growth factor-β; PPARα, peroxisome proliferator-activated receptor α; RXR, retinoid X receptor; CKD, chronic kidney disease; CRY1, cryptochrome circadian regulator 1; Exo-miR-26a, exosome-encapsulated miR-26a; TLR, toll-like receptor; NF-κB, nuclear factor-κB

As key posttranscriptional regulators of gene expression, RNA modifications influence various biological processes and disease progression. Small-molecule inhibitors targeting specific “writers” or “erasers” of RNA modifications, such as m^6^A, m^5^C, and Ψ, are promising for CKD treatment. For example, the small-molecule inhibitor STM2457, which targets RNA methyltransferase METTL3, binds to METTL3 and inhibits its methyltransferase activity, thereby alleviating renal fibrosis in vivo [82]. IOX1, an inhibitor of the m^6^A demethylase ALKBH5, promotes m^6^A modification of CCL28 mRNA, enhances its stability, modulates the regulatory T cell (Treg)/inflammatory cell axis, and protects against kidney injury [136]. Another important m^6^A demethylase, FTO, removes m^6^A from single-stranded RNA via oxidative demethylation. Studies have demonstrated that its 2 inhibitors, MA2 and FB23-2, have some effects on kidney disease; additional studies are needed to clarify whether these effects are beneficial or detrimental [90,137]. The small-molecule inhibitor RG108, initially developed as a DNMT inhibitor, also inhibits the m^5^C writer NSUN2, markedly reducing renal inflammation and injury [86]. Additionally, the activity of the TET protein family—erasers of m^5^C—may be suppressed by 2,4-dinitrophenol, which potentially acts as a TET small-molecule inhibitor. Table 4 summarizes the RNA modification-related strategies for CKD treatment.

**Table 4.** Strategies for CKD treatment related to RNA modifications

Reagents	Models	Mechanisms	**References**
STM2457	UUO mouse model	METTL3-specific inhibitor	[82]
MA2	High glucose-cultured HK-2 cells	m^6^A demethylase FTO inhibitor (decreased HDAC5 expression)	[90]
FB23-2	SD rats administered intragastrically with QTXZG decoction	m^6^A demethylase FTO inhibitor	[137]
IOX1	I/R injury mouse model	m^6^A demethylase ALKBH5 inhibitor	[136]
RG108	Cisplatin- and hypoxia–reoxygenation-induced AKI	Inhibited NSUN2	[86]

UUO, unilateral ureteral obstruction; MA, meclofenamic acid; FTO, fat mass and obesity-associated protein; HDAC5, histone deacetylase 5; METTL3, methyltransferase-like 3; ALKBH5, AlkB homolog 5; NSUN2, NOP2/Sun RNA methyltransferase family member 2; SD, Sprague–Dawley; QTXZG, Qiteng Xiaozhuo granules; I/R, ischemia–reperfusion injury

Future gene therapy approaches may offer promising avenues for combating ferroptosis in patients with CKD. Specifically, the delivery of functional copies of genes, such as GPX4 or SLC7A11, to renal cells may help restore normal antioxidant defenses and suppress ferroptosis [138]. This gene-based approach directly addresses the core mechanism of ferroptotic injury in CKD. However, this strategy faces substantial challenges, including efficient gene delivery, tissue specificity, and long-term stability of the transduced genes. In parallel, we propose a potential combination strategy that integrates gene therapy with epigenetic modulation, targeting the repression of ferroptosis-protective genes. For example, small-molecule inhibitors that disrupt the interaction between methyl-CpG-binding proteins and HDACs could be employed to relieve transcriptional silencing and enhance endogenous antioxidant defenses [139]. Strategies that target ferroptosis through GPX4 activation or iron chelation could be synergistically combined with epigenetic modulators to achieve better regulation of ferroptosis-related gene expression in CKD [94]. Additionally, targeting chromatin remodeling complexes or epigenetic interaction processes with small-molecule inhibitors, such as FHD-286 and iCARM1, holds promise for reversing fibrosis and inflammation in CKD [43].

Recent studies have elucidated the role of 3-dimensional (3D) epigenetic mechanisms in ferroptosis, suggesting that the 3D chromatin architecture modulates gene expression by influencing chromatin accessibility and long-range interactions among regulatory elements, such as enhancers and promoters [140]. Advanced 3D genomics technologies such as Hi-C and HiChIP have revealed the critical role of chromatin spatial architecture in regulating biological processes, including ferroptosis. Although the 3D epigenetic mechanisms underlying ferroptosis remain incompletely characterized, their links to disease mechanisms and clinical applications are increasingly recognized. However, despite these innovative therapeutic directions, several critical challenges hinder clinical translation. These include achieving efficient, cell type-specific gene delivery; ensuring the long-term expression and safety of transduced genes; and overcoming off-target effects and toxicity associated with epigenetic therapies. Therefore, restoring gene- or organ-specific epigenetic profiles and designing more precise, targeted therapies may offer promising approaches to slow or even reverse CKD progression. One promising approach is to integrate kidney-specific promoters into gene therapy vectors to reduce off-target effects [141]. Conjugating antibodies that specifically recognize kidney diseases markers (such as podocyte- or proximal renal tubular cell-specific antigens) with epigenetic drugs to directly target renal cells is also a powerful strategy for targeted drug delivery [142]. The development of kidney-targeted small-molecule prodrugs, activated specifically in renal tissues to release active epigenetic drugs locally and minimize systemic side effects, is another avenue worth exploring [143]. Furthermore, exosomes derived from renal cells can efficiently deliver epigenetic drugs or gene-silencing RNAs while minimize off-target effects.

## Outlook

**Fig. 7.**
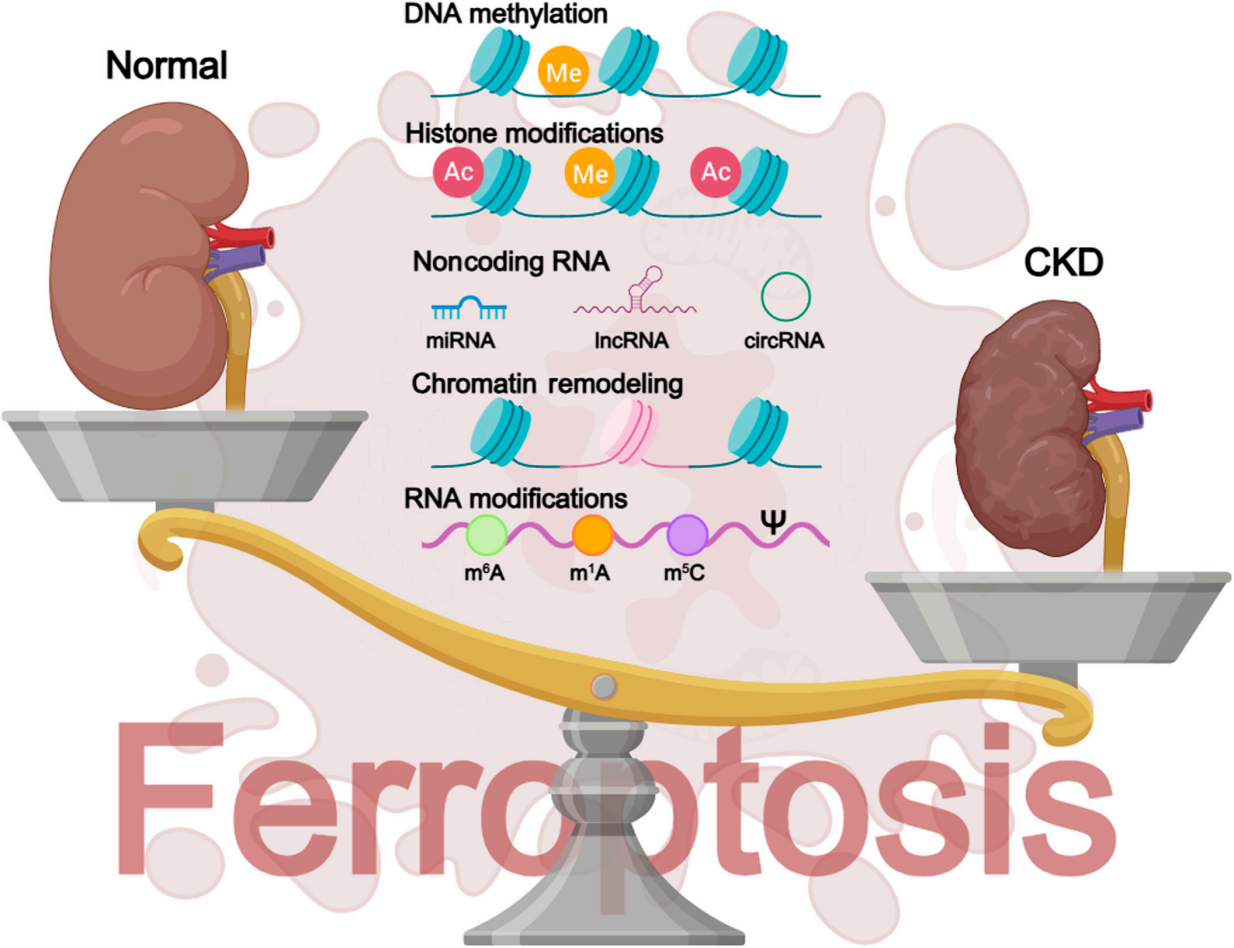
Overview diagram of the epigenetic regulation of ferroptosis in CKD. The figure was created with MedPeer.

CKD is a complex and progressive condition that imposes a heavy burden on global health. Current treatments are limited in their capacity to slow disease progression and prevent the onset of ESRD. Recent studies have emphasized the involvement of ferroptosis in CKD pathogenesis. Epigenetic modifications, such as DNA methylation, histone modifications, and noncoding RNA-mediated regulation, also play crucial roles in CKD progression by modulating gene expression without altering DNA sequences [10]. This review examines the intersection of ferroptosis and epigenetic regulation in CKD (Fig. 7), sheds new light on the mechanisms underlying disease progression, and identifies potential therapeutic targets.

The key mechanisms underlying ferroptosis encompass dysregulation of iron metabolism, lipid peroxidation cascades, dysfunction of antioxidant systems, and mitochondrial damage [4]. Crucially, iron metabolism is essential for cellular function; its disruption elevates intracellular labile iron levels, thereby triggering ferroptosis. Lipid peroxidation, particularly of PUFAs, disrupts cell membrane structure and function, contributing to CKD progression. The antioxidant system, consisting of enzymes (such as GPX4) and nonenzymatic antioxidants (such as GSH), is vital for combating oxidative stress and preventing ferroptosis. Mitochondrial dysfunction, marked by morphological alterations and impaired function, exacerbates both oxidative damage and ferroptosis.

Epigenetic modifications, including DNA methylation, histone modifications, and noncoding RNA-mediated regulation, can influence the expression of genes related to ferroptosis [7]. DNA methylation can repress or activate genes, whereas histone modifications can alter chromatin structure, thereby affecting gene transcription. Noncoding RNAs, such as miRNAs, lncRNAs, and circRNAs, modulate gene expression epigenetically, influencing cellular functions and disease progression. These epigenetic modifications can mediate the modulation of genes involved in iron metabolism, lipid peroxidation, and antioxidant defense pathways, thereby regulating ferroptosis in CKD.

This review also highlights the therapeutic potential of targeting ferroptosis and epigenetic modifications in CKD. Strategies to mitigate ferroptosis-mediated kidney injury include blocking lipid peroxidation and ROS accumulation, using iron chelators, enhancing GPX4 activity or up-regulating SLC7A11 expression, and targeting Nrf2-activating compounds. Epigenetic therapies, including DNMT inhibitors, HMT inhibitors, and HDAC inhibitors, can reverse abnormal epigenetic patterns in CKD. The bidirectional regulation of ferroptosis by DNA methylation (such as hypermethylation of the GPX4 promoter) and histone modifications (such as H3K27ac) suggests a “ferro-epigenomic code” that governs disease severity. Advanced techniques, such as CUT&Tag for chromatin accessibility mapping and metabolic tracing of oxidized lipids, could reveal how epigenetic landscapes dynamically respond to ferroptotic stimuli [144]. The integration of organoid models with CRISPR interference screens may identify master regulators at the iron–lipid–epigenome interface, potentially revealing circadian-regulated vulnerabilities in CKD progression. Additionally, targeting noncoding RNAs, such as miRNA antagonists, mimics, or anti-miRNA oligonucleotides, may offer promising therapeutic strategies by blocking the functions of miRNAs that promote ferroptosis.

In addition to conventional DNMT/HDAC inhibitors, epigenetic modulation of ferroptosis requires spatiotemporal precision. Lipid nanoparticle-delivered small interfering RNAs [145] and engineered zinc-finger proteins [146] exemplify allele-specific strategies. Moreover, ferroptosis-selective proteolysis-targeting chimeras (PROTACs) degrade key effectors [147], and mitochondrion-targeted iron chelators conjugated with BH4 mimetics [148] can achieve compartmentalized artificial intelligence-driven compound libraries, which may accelerate the discovery of dual-action molecules that simultaneously suppress lipid peroxidation (such as LOX inhibitors) and activate antiferroptotic lncRNAs (such as NEAT1). However, clinical implementation faces several hurdles, including kidney-specific delivery barriers and interpatient heterogeneity in ferroptosis susceptibility. Nanotechnology-enabled renal tropism strategies, such as mesoscale nanoparticles that exploit the tubular reabsorption mechanism, could increase drug bioavailability [149]. Personalized risk stratification might integrate urinary 8-iso-PGF2α [150] (a lipid peroxidation marker) with circulating miR-214-3p [151] (an epigenetic ferroptosis driver). Furthermore, leveraging synthetic biology to design “sense-and-rescue” circuits, in which engineered cells detect early ferroptotic signals (such as elevated labile iron) and autonomously release GSH precursors, represents a frontier in closed-loop modulation. However, unintended circuit activation caused by iron homeostasis fluctuations or oxidative stress in nontarget tissues could result in excessive or misdirected release of GSH precursors. Engineered cells might also trigger immune responses or lose circuit stability over time due to mutations or environmental variability. Thus, rigorous circuit design, integration of safety switches, and thorough preclinical validation are essential for future translational applications.

In conclusion, the interplay between ferroptosis and epigenetic modifications in CKD presents a promising avenue for developing novel therapeutic approaches. Future studies should further elucidate the molecular mechanisms underlying ferroptosis and epigenetic regulation in CKD patients while investigating targeted therapies to slow disease progression and improve clinical outcomes. Furthermore, the ferroptosis–epigenetics axis may constitute a universal stress-response adaptation mechanism relevant to DN, cardiac fibrosis, and neurodegenerative disorders [10]. Cross-disease comparative studies using pancancer-like consortium datasets could uncover conserved regulatory networks. Longitudinal clinical trials combining epigenetic reprogramming (such as BET inhibitors) with ferroptosis surveillance (such as magnetic resonance imaging-based iron quantification) will be essential to validate the universality of this therapeutic paradigm. Ultimately, combining ferroptosis chronotherapy with epigenetic age reversal strategies (such as transient Yamanaka factor expression) could potentially not only halt but also reverse established renal fibrosis pathology.
