# Supplementary material for: Epigenetic Regulation of Ferroptosis in Chronic Kidney Disease: Mechanisms and Implications
Source: Research (Wash D C). 2025 Oct 9;8:0934. doi: 10.34133/research.0934 (PMC12508529; doi:10.34133/research.0934)
Supplement: Supplementary 1 — Tables S1 to S7 [file research.0934.f1.doc]

Supplementary Material

**Table 1. Search strategy: MeSH index terms**

| Rank | Search terms |
| --- | --- |
| #1 | TS = (“Ferroptosis” OR “Oxytosis” OR “Cell Death” OR “Programmed Cell Death” OR “Iron” OR “Iron Metabolism” OR “Reactive Oxygen Species” OR “ROS” OR “Lipid Peroxidation” OR “Glutathione” OR “Antioxidants” OR “Lipids” OR “Glutathione Peroxidase 4” OR “GPX4” OR “Voltage-dependent Anion Channels” OR “VDAC2/3” OR “Transferrin Receptor 1” OR “TFR1” OR “NADPH oxidase” OR “NOX” OR “SLC7A11” OR “Cysteinyl-tRNA Synthetase” OR “CARS” OR “Nuclear Factor-erythroid 2-related Factor 2” OR “Nrf2” OR “RSL3” OR “Ferrostatin-1” OR “Fer-1” OR “Liproxstatin-1” OR “Lip-1”) |
| #2 | TS = (“Epigenetic Modification” OR “Epigenesis” OR “Genetic” OR “Epigenetics” OR “DNA Methylation” OR “Histone Modification” OR “Histone Methylation” OR “Histone Acetylation” OR “Histone Phosphorylation” OR “Chromatin” OR “Chromatin Remodeling” OR “Gene Expression Regulation, Epigenetic” OR “Non-coding RNA” OR “RNA Methylation” OR “m6A Methylation” OR “Transcription” OR “DNA Methyltransferases” OR “Histone Methyltransferases” OR “Histone Demethylases” OR “Histone Deacetylases” OR “Histone Acetyltransferases” OR “DNA-Binding Proteins” OR “Epigenetic Reprogramming” OR “CpG Islands”) |
| #3 | TS = (“Chronic Kidney Disease” OR “Chronic Kidney Failure” OR “Renal Insufficiency” OR “Kidney Failure” OR “Renal Failure” OR “Proteinuria” OR “Uremia” OR “Renal Dialysis” OR “Renal Anemia” OR “Renal Replacement Therapy” OR “End-Stage Kidney Disease” OR “End-Stage Renal Disease” OR “ESRD”) |

**Table 2. The top 10 most productive countries**

| Rank | Country | Documents | Citations | Total link strength |
| --- | --- | --- | --- | --- |
| 1 | The United States | 246 | 16449 | 144 |
| 2 | China | 234 | 7453 | 61 |
| 3 | Japan | 88 | 7794 | 45 |
| 4 | Italy | 43 | 1475 | 47 |
| 5 | Germany | 41 | 3666 | 58 |
| 6 | United Kingdom | 39 | 1946 | 56 |
| 7 | Sweden | 31 | 4136 | 51 |
| 8 | France | 30 | 1182 | 35 |
| 9 | Spain | 29 | 910 | 11 |
| 10 | Brazil | 27 | 888 | 22 |

**Table 3. The top 10 most productive organizations**

| Rank | Organization | Documents | Citations | Total link strength |
| --- | --- | --- | --- | --- |
| 1 | Karolinska Institute | 26 | 3947 | 30 |
| 2 | The University of Tokyo | 18 | 1451 | 9 |
| 3 | Tohoku University | 14 | 1527 | 6 |
| 4 | University of Glasgow | 13 | 1235 | 17 |
| 5 | Shanghai Jiao Tong University | 12 | 995 | 22 |
| 6 | Southern Medical University | 12 | 529 | 14 |
| 7 | Shandong University | 12 | 176 | 7 |
| 8 | Fudan University | 11 | 717 | 23 |
| 9 | Zhejiang University | 11 | 767 | 18 |
| 10 | Harvard Medical School | 11 | 478 | 11 |

**Table 4. The top 10 most citation journal sources**

| Rank | Source | Documents | Citations | Total link strength |
| --- | --- | --- | --- | --- |
| 1 | Kidney International | 22 | 2389 | 105 |
| 2 | International journal of molecular sciences | 21 | 696 | 56 |
| 3 | Antioxidants | 20 | 544 | 53 |
| 4 | Journal of the American Society of Nephrology | 19 | 1645 | 26 |
| 5 | American Journal of Physiology - Renal Physiology | 16 | 832 | 9 |
| 6 | Nephrology Dialysis Transplantation | 14 | 901 | 44 |
| 7 | PLOS ONE | 14 | 574 | 6 |
| 8 | Scientific Reports | 13 | 420 | 23 |
| 9 | Oxidative Medicine and Cellular longevity | 12 | 1200 | 15 |
| 10 | Biomedicine & Pharmacotherapy | 12 | 218 | 12 |

**Table 5. The top 10 most productive authors**

| Rank | Author | Documents | Citations | Total link strength |
| --- | --- | --- | --- | --- |
| 1 | Peter Stenvinkel | 20 | 1111 | 40 |
| 2 | Denise Mafra | 15 | 473 | 41 |
| 3 | Masayuki Yamamoto | 11 | 1263 | 28 |
| 4 | Paul G. Shiels | 11 | 528 | 22 |
| 5 | Masaomi Nangaku | 11 | 561 | 18 |
| 6 | Tetsuhiro Tanaka | 10 | 576 | 18 |
| 7 | Nosratola D. Vaziri | 10 | 1063 | 17 |
| 8 | Ludmila F. M. F. Cardozo | 8 | 218 | 29 |
| 9 | Norio Suzuki | 7 | 515 | 17 |
| 10 | Reiko Inagi | 7 | 325 | 14 |

**Table 6. The top 20 most frequently occurring keywords**

| Rank | Keyword | Occurrences | Total link strength |
| --- | --- | --- | --- |
| 1 | Oxidative Stress | 137 | 226 |
| 2 | Chronic Kidney Disease | 131 | 214 |
| 3 | Inflammation | 76 | 155 |
| 4 | Nrf2 | 60 | 96 |
| 5 | Diabetic Nephropathy | 56 | 63 |
| 6 | Reactive Oxygen Species | 40 | 47 |
| 7 | Acute Kidney Injury | 36 | 58 |
| 8 | Kidney | 26 | 36 |
| 9 | Apoptosis | 26 | 31 |
| 10 | Anemia | 23 | 59 |
| 11 | Diabetic Kidney Disease | 22 | 24 |
| 12 | Iron | 21 | 41 |
| 13 | Mitochondria | 21 | 28 |
| 14 | CKD | 20 | 37 |
| 15 | Autophagy | 20 | 31 |
| 16 | Ferroptosis | 20 | 18 |
| 17 | Renal Fibrosis | 19 | 26 |
| 18 | Vascular Calcification | 19 | 14 |
| 19 | Erythropoietin | 18 | 39 |
| 20 | Fibrosis | 18 | 26 |

**Table 7. The top 10 most co-cited references**

| Rank | First Author | Title | Journal | Year | Citations |
| --- | --- | --- | --- | --- | --- |
| 1 | Pablo E. Pergola | Bardoxolone Methyl and Kidney Function in CKD with Type 2 Diabetes | New Engl J Med | 2011 | 66 |
| 2 | Dick de Zeeuw | Bardoxolone Methyl in Type 2 Diabetes and Stage 4 Chronic Kidney Disease | New Engl J Med | 2013 | 62 |
| 3 | Stacey Ruiz | Targeting the transcription factor Nrf2 to ameliorate oxidative stress and inflammation in chronic kidney disease | Kidney Int | 2013 | 59 |
| 4 | Manchang Liu | Transcription factor Nrf2 is protective during ischemic and nephrotoxic acute kidney injury in mice | Kidney Int | 2009 | 56 |
| 5 | Hyun Ju Kim | Contribution of impaired Nrf2-Keap1 pathway to oxidative stress and inflammation in chronic renal failure | Am J Physiol-Renal | 2010 | 40 |
| 6 | Tao Jiang | The protective role of Nrf2 in streptozotocin-induced diabetic nephropathy | Diabetes | 2010 | 37 |
| 7 | Masahiro Nezu | Transcription factor Nrf2 hyperactivation in early-phase renal ischemia‒reperfusion injury prevents tubular damage progression | Kidney Int | 2017 | 34 |
| 8 | Hongting Zheng | Therapeutic potential of Nrf2 activators in streptozotocin-induced diabetic nephropathy | Diabetes | 2011 | 33 |
| 9 | K Itoh | An Nrf2/small Maf heterodimer mediates the induction of phase II detoxifying enzyme-encoding genes through antioxidant response elements | Biochem Bioph Res Co | 1997 | 33 |
| 10 | Pablo E Pergola | Effect of bardoxolone methyl on kidney function in patients with T2D and Stage 3b-4 CKD | Am J Nephrol | 2011 | 32 |
